# Predictors of Mortality in Patients With Interstitial Lung Disease Treated With Corticosteroids: Results from a Cohort Study

**DOI:** 10.1097/MD.0000000000000175

**Published:** 2014-12-05

**Authors:** Kiyoshi Migita, Toru Arai, Yuka Jiuchi, Yasumori Izumi, Nozomi Iwanaga, Chieko Kawahara, Eiichi Suematsu, Tomoya Miyamura, Hiroshi Tsutani, Yojiro Kawabe, Ryutaro Matsumura, Shunsuke Mori, Shiro Ohshima, Shigeru Yoshizawa, Yasuo Suenaga, Fumitaka Ogushi, Masaharu Kawabata, Hiroshi Furukawa, Toshihiro Matsui, Seiji Bito, Shigeto Tohma

**Affiliations:** From the Japanese National Hospital Organization (NHO)—EBM study group for Adverse Effects of Corticosteroid therapy (J-NHOSAC), Meguro, Tokyo (KM, TA, ES, TM, HT, YK, RM, SM, SO, SY, YS, FO, MK, HF, TM, SB, ST); and Department of Rheumatology and General Internal Medicine Nagasaki Medical Center, Omura, Nagasaki, Japan (KM, YJ, YI, NI, CK).

## Abstract

Interstitial lung disease (ILD) has a heterogeneous clinical presentation and establishing prognosis for these patients is challenging. We investigated the clinical characteristics and outcome of patients with idiopathic interstitial pneumonias (IIPs) and patients with connective tissue disease-associated interstitial lung disease (CTD-ILD). We conducted a multicenter prospective study on 104 patients diagnosed with IIPs and 29 patients diagnosed with CTD-ILD, which were newly diagnosed and treated with corticosteroids initially. We compared the clinical characteristics, high-resolution computed tomography (HRCT) imaging date, and outcomes. Cox proportional hazard regression analysis was used to identify variables with increased risk of death. Survival was analyzed according to the Kaplan–Meier method and was assessed with the log-rank test. Of 133 patients with IIPs (n = 104) or CTD-ILD (n = 29), 44 patients died during the follow-up period (mean: 1.6 ± 0.78 years). Patients with IIPs seemed to be associated with worse survival compared with those with CTD-ILD; however, this difference was not significant (log-rank test, *P* = 0.084). Significant predictors for mortality in patients with IIPs at baseline were lower for performance status and definite usual interstitial pattern (UIP) on HRCT. Patients with UIP experienced worse survival than those with non-UIP. A definite UIP on HRCT and lower baseline performance status have important prognostic implications in patients with IIPs.

## INTRODUCTION

Interstitial lung disease (ILD) is a progressive fibrotic disease of the lung parenchyma that includes a broad spectrum of disorders that vary in their clinical presentation, natural history, disease, prognosis, and treatment.^1,2^

Therapeutic options are currently limited and predictive markers are inadequate. Histological subtype is the strongest prognostic marker for ILD, but whether this applies across all subtypes, including connective tissue disease-associated interstitial lung disease (CTD-ILD), is debatable.^3^ The American Thoracic Society (ATS) and the European Respiratory Society (ERS) provided a consensus classification of the idiopathic interstitial pneumonias (IIPs).^4^ The IIPs are currently classified into 6 entities, which differ not only in disease, but also in radiologic and clinical features, including prognosis.^5^ Of these, the 2 most common histological patterns are usual interstitial pneumonia (UIP) and nonspecific interstitial pneumonia (NSIP).^6^ UIP is predominantly idiopathic pulmonary fibrosis (IPF), and NSIP is a more heterogeneous group clinically.^7^ Recent studies have shown that patients with IPF were found to have much worse outcome compared with patients with CTD-ILD.^8^ The NSIP has been reported as the major histologic pattern in CTD.^9^ Further, it has been reported that patients with CTD-ILD have a better prognosis than patients with IIPs.^10^

Epidemiological studies on ILD are relatively scarce, but the existing studies show that there are wide variations in the outcomes of the various types of ILD. In the present study, we performed a multicenter, cohort study of newly diagnosed ILD using the Japanese National Hospital Organization (NHO) database as described previously.^11^ In the present study, we prospectively analyzed 133 consecutive patients with newly diagnosed ILD, which were treated with glucocorticoids to identify predictive factors of serious adverse events. The second purpose of this study was to investigate whether there was a difference in the prognosis of patients with CTD-ILD compared with those with IIPs.

## METHODS

### Study Design

We previously conducted a multicenter cohort study on patients with recently diagnosed autoimmune disease in NHO hospitals (a total of 55 hospitals). Patients were eligible if they were initially treated with glucocorticoids against the following autoimmune diseases, which were newly diagnosed (within the 4 weeks before the entry) by the established criteria.^11^ A total of 604 patients with newly diagnosed autoimmune disease were enrolled between April 1, 2006, and March 31, 2008, and regularly followed concerning the occurrence of glucocorticoid-related adverse effects. The study was approved by the ethical committee of the NHO central internal review board (No. 0512014, 2006). Written informed consent was obtained from each individual. Using this database, we performed a subanalysis of the enrolled patients with ILD. A standard form was used to collect clinical information, including symptoms, smoking history, medication use, environmental history, family history, and physical findings, high-resolution computed tomography (HRCT), and pulmonary function tests. Data from all participating physicians were entered into the J-NHOSAC database at the data center of the International Medical Center of Japan in Tokyo, Japan via the HOSPnet Internet system.

### Diagnostic Criteria

A diagnosis of ILD was established according to the criteria of the ATS, including consistent clinical features and pulmonary function tests, radiographic evidence of interstitial disease, and/or lung histopathology consistent with this diagnosis.^5^ All patients were evaluated for ILD using HRCT scans of their lungs. Diagnosis of ILD was determined by a panel of ILD expert clinicians and chest radiologists based on serology, clinical signs, and HRCT analysis. IPF/UIP was diagnosed according to the ATS/ERS consensus classification, and the UIP was determined at the biopsy.^12^ Alternatively, UIP was diagnosed by HRTC when the following criteria were fulfilled. The UIP is characterized by all 4 features of the disease, which include basal and subpleural predominance; reticular pattern, with associated traction bronchiectasis; honeycombing appearance; and absence of features listed as inconsistent with a UIP.^12^

CTD-ILD was diagnosed if ILD was found in the presence of rheumatic disease. The diagnosis of various CTDs were determined by the treating rheumatologist and confirmed by medical record review. Patients were classified as having systemic sclerosis, rheumatoid arthritis, systemic lupus erythematosus, or Sjögren syndrome based on the American College of Rheumatology criteria. Bohan and Peter^13^ criteria were applied for the diagnosis of inflammatory myopathies, including polymyositis and dermatomyositis. The diagnosis of mixed connective disease was based on the clinical features described by Sharp et al.^14^

### Outcome Variables

At the initiation of the study, standardized lists were used to document adverse events (AEs), which were classified using the System Organ Class (SOC) of the Medical Dictionary for Regulatory Activities (MedDRA; version 11.1). Patients were followed up every 3 months by the chief physician for each of the NHO hospitals, who collected clinical findings (disease activity, severity, performance status, blood pressure, and body weight) and laboratory data (complete blood cell count, biochemistry, and urinalysis). All physicians documented episodes of infection requiring medical care and death certificates and the causes of deaths that occurred during the follow-up periods.

### Follow-Up Data

Patients were followed up every 3 months by the chief physician for each of the NHO hospitals, who collected clinical findings (disease activity, severity, performance status, blood pressure, and body weight) and laboratory data (complete blood cell count, biochemistry, and urinalysis). The telephone interview concerning the health assessment and the presence of glucocorticoids (GCs)-related AEs was conducted against few patients who were moved or transferred to another hospital at the end of cohort. However, overall outcome was not available from 2 patients (2/133, 1.5%) at the end of study. In statistical analysis, we excluded these participants without final outcome data.

### Medications

Details of GCs, immunosuppressants, and biologics were recorded at each visit, including the route of administration and dose. We categorized GC exposure according to the mean daily dose throughout the follow-up period for each patient. We calculated “dose equivalents” of prednisolone as follows: 1 mg of prednisolone = 5 mg of cortisone = 4 mg of hydrocortisone = 1 mg of prednisone = 0.8 mg of triamcinolone = 0.8 mg of methylprednisolone = 0.15 mg of dexamethasone = 0.15 mg of betamethasone.^15^

### Statistical Analysis

All categorical variables were reported as frequency (percentages). Qualitative variables were compared using the chi-square test (or Fisher exact test when appropriate), and quantitative variables were compared using the Mann–Whitney *U* test. Cox proportional hazard models were used to estimate the risk of mortality. In Cox proportional hazard models, we identified the best subset of explanatory variables by all combination as variable selection in terms of score statistics. Results are expressed as hazard ratios with 95% confidence intervals. Survival, related to follow-up time, was analyzed using the Kaplan–Meier method and compared using the log-rank test. Two-sided *P* values <0.05 were considered statistically significant. All statistical assessments were performed using the SAS software, Version 9 (SAS Institute Inc, Cary, NC).

## RESULTS

### Baseline Characteristics

The study cohort comprised of 133 patients with ILD comprised. All patients met the criteria for ILD. Of the 133 patients, 104 had IIPs and 29 had CTD-ILD. The baseline clinical and laboratory features are summarized in Table 1. The mean age of patients at baseline was 68.6 ± 11.4 years and 45.1% of patients were female. The mean follow-up period was 19.4 months. The primary diseases of CTD-ILD patients (n = 29) were rheumatoid arthritis (n = 12), systemic sclerosis (n = 2), polymyositis (n = 2), dermatomyositis (n = 4), Sjögren syndrome (n = 3), vasculitis syndrome (n = 4), Bechet disease (n = 1), and undifferentiated connective tissue disease (n = 1). Arterial blood gas values were all similar among the 2 groups. Twenty-four of 133 patients were classified histologically as UIP (n = 15), NSIP (n = 7), lymphocytic interstitial pneumonia (n = 1), and organizing pneumonia (n = 1) according to the lung biopsy results. Of the remaining 109 patients, 43 were classified as having UIP pattern and 66 had non-UIP pattern by the HRCT scan imaging and according to the ATS/ERS criteria.^12^

**TABLE 1:**
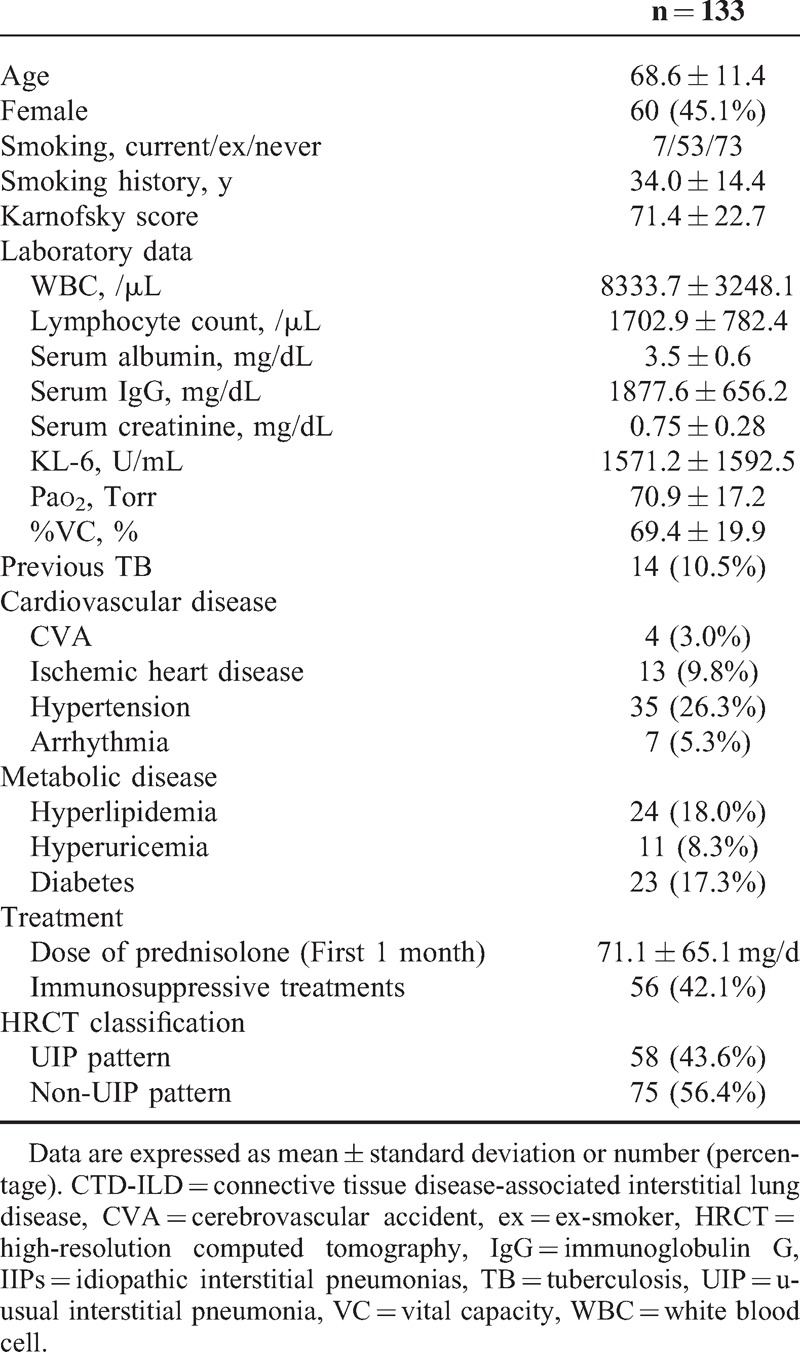
Baseline Clinical and Demographic Features of all Patients

### Comparisons of CTD-ILD and IIPs

Comparisons of demographic and clinical characteristics among patients with IIPs and CTD-ILD are shown in Table 2. Comparison of baseline clinical features revealed that patients with CTD-ILD (n = 29) were younger and more often female at enrollment than patients with IIPs. Regarding treatment, all patients were treated with corticosteroids at baseline, and the mean dose of corticosteroid for the first 1 month was 75.5 ± 68.3 mg/d in patients with IIPs and 55.6 ± 50.3 mg/d in patients with CTD-ILD. Of the total patients, 77 patients (57.9%) were treated with corticosteroids alone, and 56 patients (42.1%) were treated with corticosteroids and immunosuppressive agents. These immunosuppressive agents included cyclosporine A (n = 32), cyclophosphamide (n = 23), and azathioprine (n = 8). More patients with IIPs presented a UIP (51/104, 49.0%) compared with those with CTD-ILD (7/29, 24.1%) on HRCT analysis (*P* = 0.017).

**TABLE 2:**
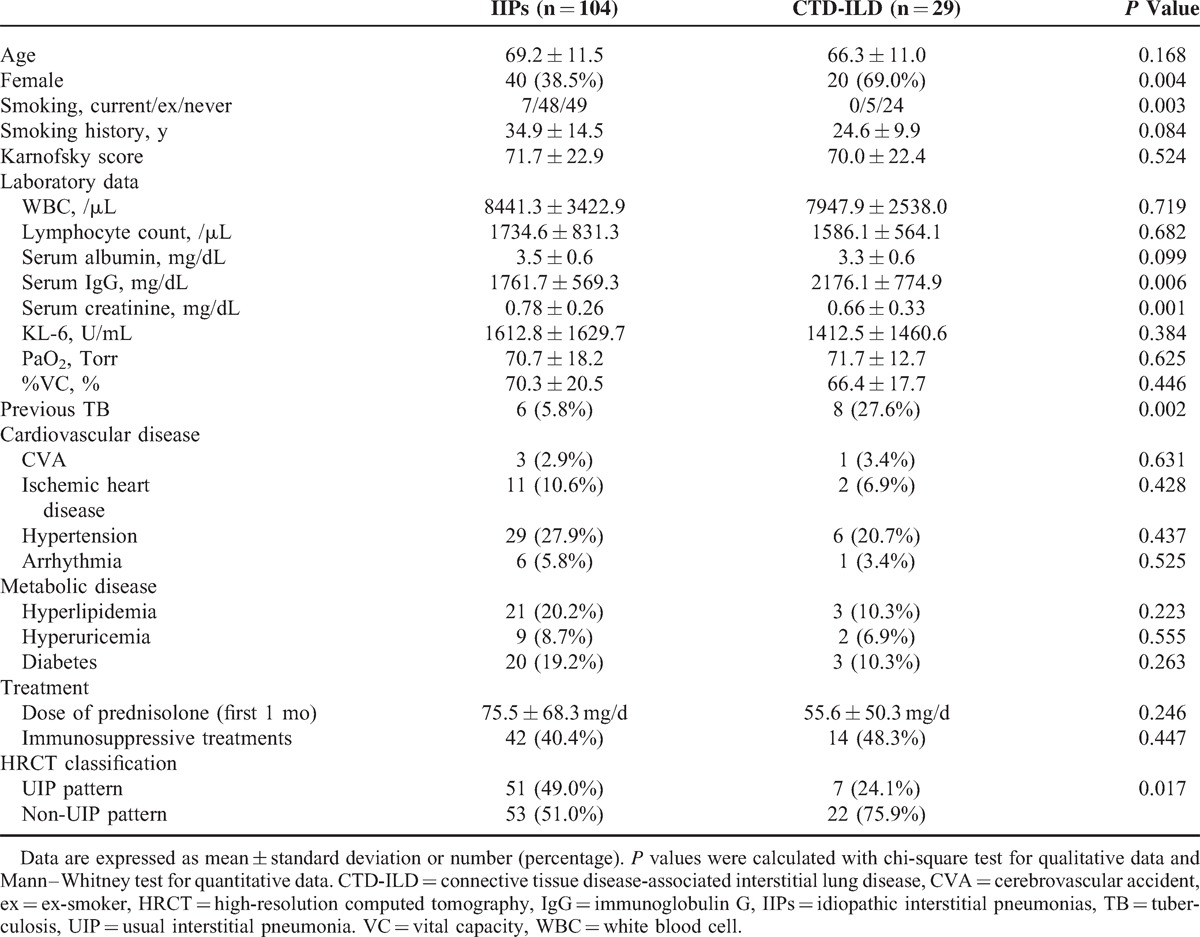
Baseline Clinical and Demographic Features of Patients With IIPs and CTD-ILD

### Survival and Causes of Death

**FIGURE 1:**
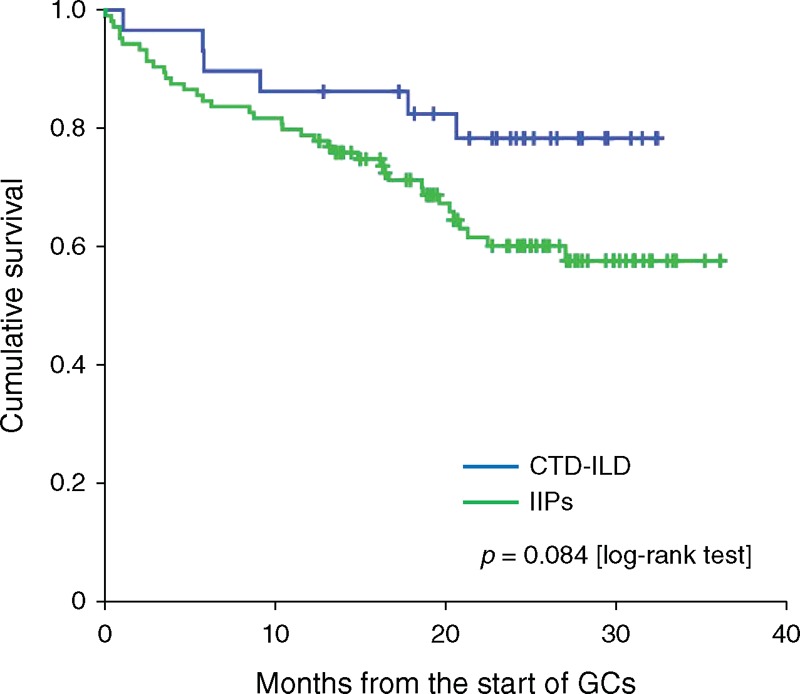
Kaplan–Meier survival curves of patients with IIPs and CTD-ILD. Statistically significant difference was not observed between patients with IIPs and CTD-ILD (*P* = 0.084, log-rank test). However, patients with IIPs seemed to be associated with worse survival compared with those with CTD-ILD. CTD-ILD = connective tissue disease-associated interstitial lung disease, GC = glucocorticoids, IIPs = idiopathic interstitial pneumonias.

Of the total 133 patients (104 IIPs and 29 CTD-ILD), 44 patients (33.1%) died during the study period with a mean follow-up time of 19.4 months. The most common causes of death were disease progression and infections including pneumonia (Table 3). As shown in Figure 1, patients in the CTD-ILD group survived longer than those in the IIP group; however, this difference was not significant (log-rank, *P* = 0.084).

**TABLE 3:**
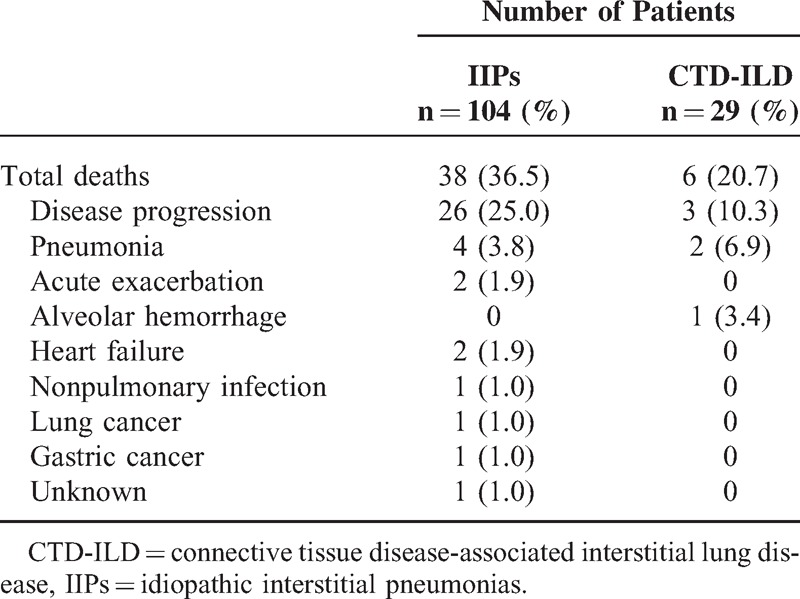
Causes of Death in Patients With Interstitial Pneumonia

### Prognostic Factors of Survival in Patients With ILD

The univariate analysis isolated several variables, which are significantly associated with fatal outcome (Table 4). Multivariate Cox regression modeling was performed to evaluate for significant predictors of mortality, adjusting for confounding variables (Table 5). The factors that were independently associated with fatal outcome include lower performance status (Karnofsky score >70) and the presence of a UIP on HRCT.

**TABLE 4:**
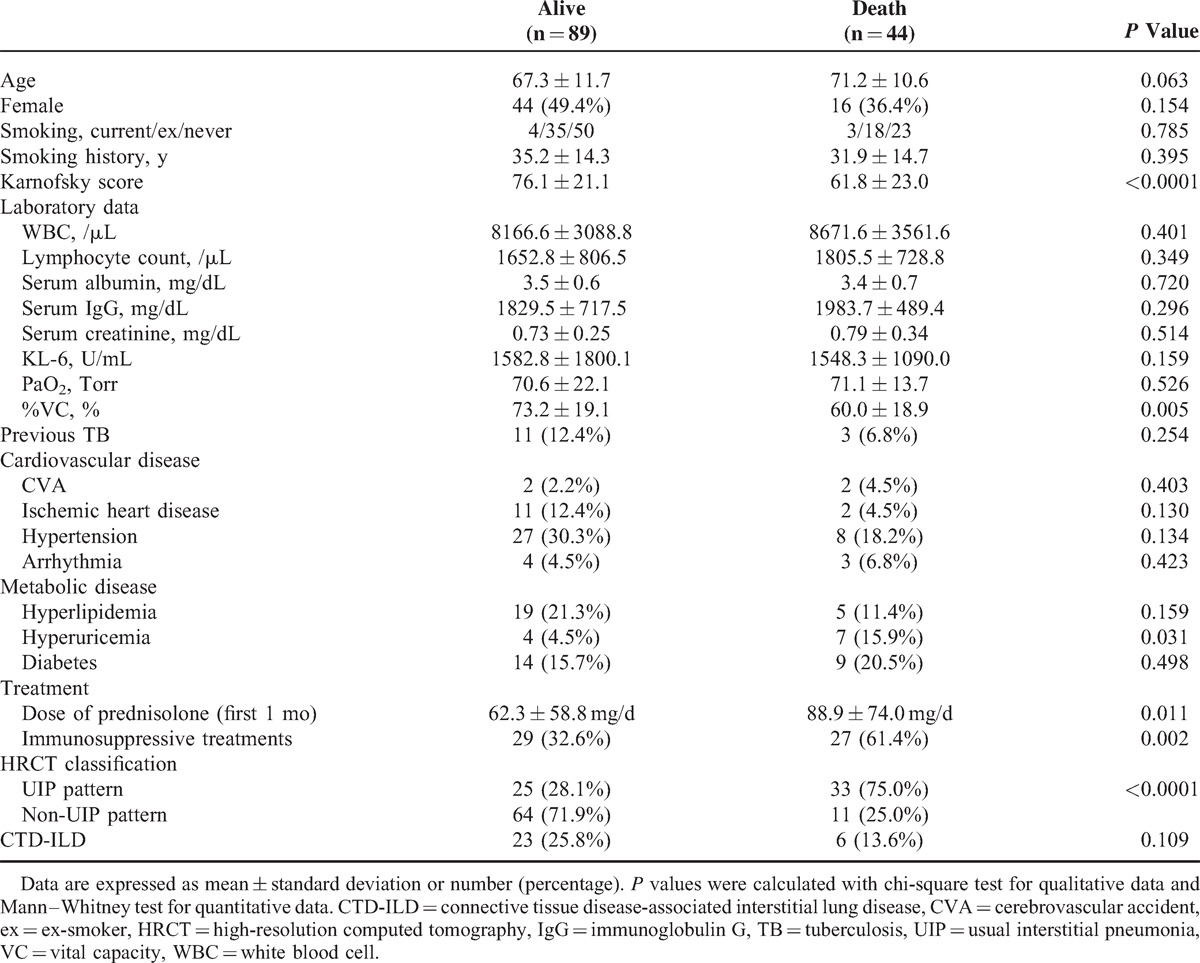
Baseline Clinical and Demographic Features of All Patients

**TABLE 5:**
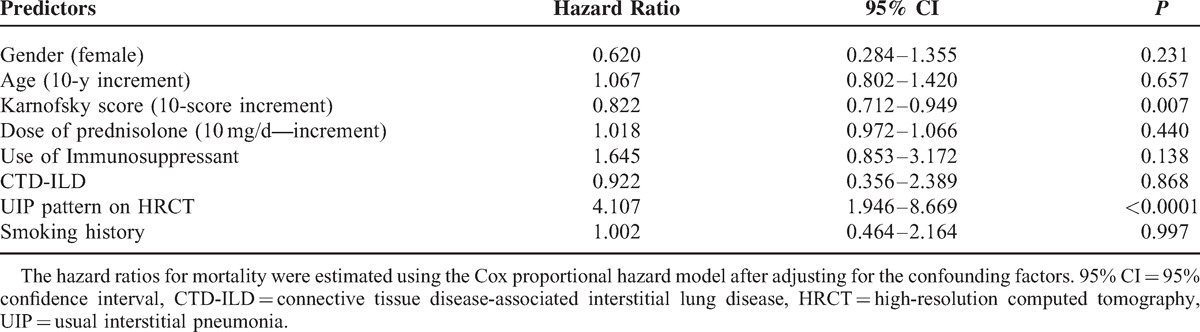
Predictors of Mortality Identified in the Multivariate Model

### Comparison of Survival of Patients With UIP and Those With A Non-UIP

**FIGURE 2:**
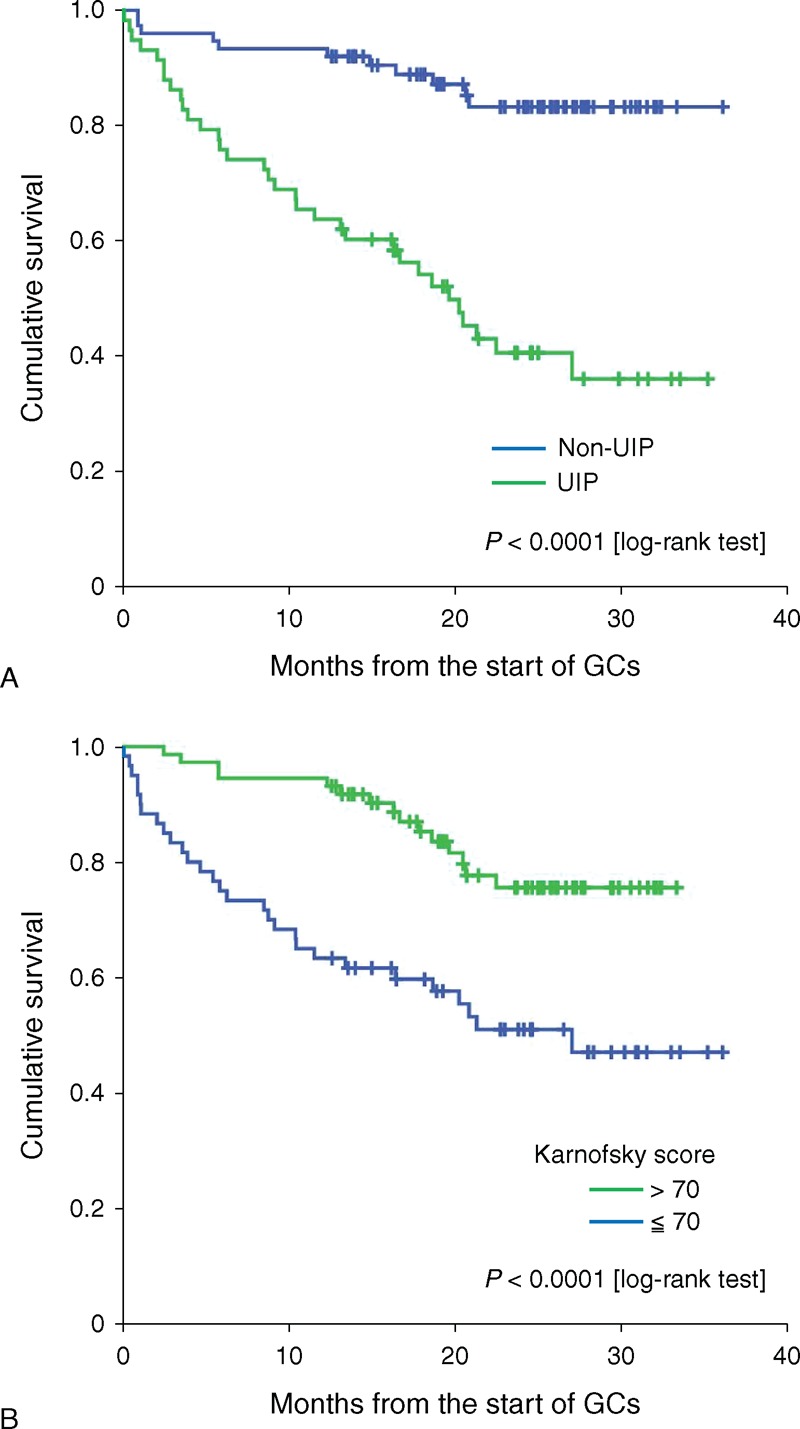
Kaplan–Meier curves of survival of patients with or without UIP. Curves are stratified by the presence or absence of UIP pattern on HRCT analysis. Statistically significant differences were observed between patients with or without UIP (*P* < 0.0001, log-rank test) (A). Kaplan–Meier curves of survival of patients stratified by performance status. Curves are stratified by baseline performance status (Karnofsky score >70, vs Karnofsky score ≤70). Statistically significant differences were observed between these 2 groups (*P* < 0.0001, log-rank test) (B). GC = glucocorticoids, UIP = usual interstitial pneumonia.

In Kaplan–Meier survival curves stratified by the category of HRCT pattern, a statistically significant difference was observed between patients with UIP and those with a non-UIP (Figure 2A). Patients with UIP on HRCT had worse survival than those with a non-UIP (log rank, *P* < 0.0001). Similarly, as shown in Figure 2B, patients with lower performance status (Karnofsky score ≤70) had worse survival than those with a higher performance status (Karnofsky score >70) (log rank, *P* < 0.0001).

## DISCUSSION

In this study, we evaluated the longitudinal prognosis of patients with newly diagnosed ILD. Our study results indicated that ILD patients with UIP pattern on HRCT had worse prognosis than did ILD patients with non-UIP pattern, mainly owing to the disease progression of ILD or associated infections during the course of corticosteroid treatments. Recent large scale of studies indicated that age, gender, and physiological parameters were significant prognostic factor in patients with ILD.^16–18^ However, these factors had not been isolated as predictors for mortality in our study. The limited numbers of participants and insufficiency of pulmonary function tests in our study may contribute to these discrepancies.

ILD represents a heterogeneous group of diseases that involves inflammation and interstitial fibrosis of the lung parenchyma.^19^ The most common type of ILD includes IIPs and CTD-ILD. Among patients with IIPs, HRCT and histopathological patterns have been shown to be of important prognostic significance.^3^ Most notably, the UIP found in patients with IPF is associated with poor outcomes.^20^ Further, it has been reported that initial HRCT findings have prognostic significance in IIPs.^21^ The role of radiologists in the diagnosis has been continuously emphasized in recent years because of the significant contribution of HRCT to the diagnosis of IIPs. According to the 2011 evidence-based guidelines, UIP can be diagnosed by HRTC.^12^ In the present study, patients with non-UIP had better prognosis than those with typical UIP on HRCT. Our study showed that the presence of UIP predicts poor survival compared with non-UIP, which is concordant with the concept that the UIP has important prognostic implications.^7^ Although the distinction between these histologic patterns may be difficult, the clinical outcome of the corresponding lesions is very different. Therefore, it is very important to assess lung biopsies or clinical and radiographic data very carefully, especially HRCT images.

Another purpose of this study was to investigate the prognosis of the patients with ILD, including those with CTD-ILD, who were initially treated with corticosteroids. In our study, the survival of patients with CTD-ILD did not differ from that of patients with IIPs significantly; however, the analysis showed that patients with CTD-ILD had a better prognosis than those with IIPs. One of the most pressing changes needed regarding ILD is to define the nature and importance of CTD-ILD better because this condition is associated with substantial morbidity and mortality, and the determination of the prognosis still remains controversial.^22^ The reports of CTD-IP that evaluated its histological patterns showed that NSIP is more common than UIP.^23^ Although CTD-ILD has a better prognosis than IPF, it is not certain whether this is because of the predominance of a NSIP or a difference in the histological findings.^24^ Although CTD-ILD is commonly associated with NSIP,^25^ UIP is also observed. It was demonstrated that UIP in non-rheumatoid arthritis(RA)-collagen vascular disease-interstitial pneumonia(IP) (non-RA-CVD-IP) is associated with a significant better survival compared with idiopathic UIP.^3^ Therefore, the prognostic significance of UIP pattern on HRCT, which was demonstrated in our study, may differ among ILD categories and could not be applied to CTD-ILD uniformly.

Recent studies indicated that the 6-minute-walk test, which is a valid physiologic measurement to assess IPF severity, is an important measure of the prognosis of ILD.^26^ However, only a select group of the population that could walk for 6 minutes can be assessed. In our study, the survival of IIPs patients with higher Karnofsky score was significantly better compared with those with lower Karnofsky score. Our results demonstrate that Karnofsky score is also a clinically useful measure to predict the risk of mortality in patients with IIPs.

Currently, the most commonly prescribed therapy for ILD consists of systemic corticosteroids.^27^ Although corticosteroids and immunosuppressive agents are used for ILD, treatment response and prognosis vary with the association of CTD, as well as with the histopathological patterns.^28^ Further studies on these issues are needed because there is currently a paucity of data. The management of ILD is challenging. Immunosuppressive treatment is generally reserved for patients with progressive ILD, especially in patients with CTD-ILD.^29^ Taken together, prospective, randomized placebo-controlled multicenter studies are needed to establish the therapeutic strategy for patients with ILD.

Our study does have some limitations. The primary outcome assessed in this analysis was all-cause mortality, therefore, some patients may have died of causes other than end-stage ILD, including nonpulmonary complications of their underlying diseases, infections, heart disease, and cancer. The diagnosis of ILD was determined by radiographic imaging without diagnoses confirmation by lung biopsies in the majority of our patients. Identification of UIP and NSIP was based on radiographic patterns observed on HRCT, which does not always correlate reliably with histologic diagnoses. Additionally, we did not determine fibrosis severity scores. UIP can be diagnosed by HRCT when all criteria are fulfilled. However, the overlap of imaging features limits the diagnosis. The diagnosis of NSIP can be difficult because of the heterogeneity of radiological findings. Therefore, the diagnosis of UIP by HRCT may not be entirely accurate. A recent guideline recommended that the diagnostic accuracy of ILD is improved multidisciplinary discussion between pulmonologist and radiologist.^18^ Radiologist and rheumatologist were involved in the diagnostic processes of all patients with ILD in our study. However, pulmonologist participated in the evaluation of IPF diagnosis in a part of enrolled patients. Additionally, autoantibody data and pulmonary function tests were limited, and therefore, we were unable to assess the prognostic effects on the survival of ILD patients with autoantibodies. A recent IPF guideline recommended that patients with IPF should not be treated with corticosteroid.^12^ Therefore, our results cannot be applied for the prognostic evaluation of patients with IPF generally. Finally, our study lacked sufficient statistical power to compare the survival between patients with IIPs and CTD-ILD.

In conclusion, we conducted a multicenter cohort study and our results demonstrated that the prognosis of patients with ILD can be predicted by patient-specific and ILD-specific features, including performance status and the presence of UIP on HRCT imaging. These findings could be useful for the management and prognostication of patients with ILD. Larger studies are required to determine whether these or other variables could be combined to develop clinical predictions that help the management of patients with ILD.
